# Determining the Role of Influencers’ Marketing Initiatives on Fast Fashion Industry Sustainability: The Mediating Role of Purchase Intention

**DOI:** 10.3389/fpsyg.2022.940649

**Published:** 2022-06-24

**Authors:** Mengmeng Liu

**Affiliations:** Business School, University of New South Wales (UNSW), Sydney, NSW, Australia

**Keywords:** fashion industry, economic growth, marketing initiatives, impulse buying tendency, purchase intension, sustainability

## Abstract

Celebrity influence plays a significant role in fostering the consumers’ impulse buying tendency and purchase intention. In the modern advertising era, the celebrity endorsement characteristics have driven the firms’ promotion campaigns, stimulating consumer purchasing behavior through celebrity branding. The study signifies the relationship between celebrity’s traits of trustworthiness, attractiveness, credibility, and expertise influence consumers’ impulse behavior. The data was collected from the 371 customers of the fast fashion industry by using the convenient-sampling technique. SMART-PLS was used for data analysis by applying structural equation modeling. The study results show that celebrity trustworthiness, the attractiveness of a celebrity endorser, the credibility of a celebrity endorser, and celebrity expertise positively impact purchase intention and impulse buying tendency. Purchase intention plays a mediating role between independent and dependent variables.

## Introduction

Over the years, the organizations have captured the global market based on different product attributes. But, now, the constant media coverage has colossally numbed the marketing standards by hooking the consumers’ attention toward the modern branding. The brisk proliferation of digital media has considerably inspired companies to accelerate their promotional activities *via* celebrity endorsement. Indeed, these profound marketing strategies have made celebrity endorsement a vital knob, grasping the consumers’ considerations (Qureshi and Malik, 2017) concerning purchase tendencies (Lin et al., 2018).

However, in modern advertising, the impulsive buying tendency has evolved for years, thus making this unique phenomenon to increase the consumers’ gratification (Pradhan et al., 2018). The rising impulsive tendencies have maximized the individuals’ urge for the store products. Consumers’ impulsive buying tendency alludes to unplanned buying (Malter et al., 2020). It is a persuading power that makes the consumer realize the need for the textile product. In recent years, the increasing market competitiveness has made it difficult for marketers to foster consumers’ impulse buying tendencies. However, after years of constant struggle, the global textile industry has again gained height by making consumers recognize the need for fashion products.

Undoubtedly, today, the consumption of clothing items has become the norm for the young generation who uses fashion to express themselves. The increasing desire for the newest products has made the consumers indulge in impulsive buying. Notably, for investigation, this study chooses the textile fashion industry because it profoundly captures the materialistic essence of impulsive buying tendencies, satisfying the individuals’ urge for the product (Chen et al., 2021). However, today’s fashion industry is at its boom and demands effective marketing strategies to capture consumer attention. The consumer purchase intention as the outcome of spontaneous encounters encourages the consumers to realize the need for the product (Maharani et al., 2020). In particular, this escalating impulsive tendency demands that organizations adopt celebrity endorsement strategies to influence individuals’ buying intentions. In the textile industry, consumers’ choosing the high-value product needs the recommendations of some authentic source. Unlike traditional marketing, celebrity endorsement characteristics have become an essential credential for driving the consumers’ impulse behavior.

Celebrities play a vital role in boosting the sale of fashion products. Celebrity branding provides symbolic information to the consumers’, thereby assisting their buying behavior. Accordingly, the study shows that the celebrity’s desirable characteristics (i.e., trustworthiness, attractiveness, credibility, and expertise) harness the brand advertising (Lou and Yuan, 2019), thereby influencing the consumers’ decisions. Hence, the inherit literature makes it necessary for the researchers to study the endorsers’ characteristics concerning the consumer buying intention (Zhu et al., 2020).

Celebrity endorsement is an effective marketing strategy that captures the buyers’ attention toward elevating brand awareness. In this regard, celebrity trustworthiness act as a credible source that enhances the buyers’ purchase intention. Celebrity trustworthiness forms a positive customer perception, thereby making them buy the product. Given the illustration, the study shows that celebrity trustworthiness plays a critical role in fostering the consumers’ purchases (Thomas and Johnson, 2019). Trustworthiness is a significant construct that refers to the endorsers’ dignity and believability. In advertising, the perceived source trustworthy makes the consumer comply with the brand message. This effective brand promotion makes the celebrity attribute (e.g., honesty, trustworthiness) increase the efficacy of consumers’ purchases. Given the illustration, the study shows that the greater the endorsers’ trustworthiness, the more the consumers find them convincing and honest (Djafarova and Rushworth, 2017).

In particular, the celebrity persona also plays a profound role in increasing brand recognition. Celebrities are the prominent icon that influences the organizations’ success in various fields. They wind up the brand sales, thereby forming a positive brand image in the minds of the consumers. Celebrity attractiveness promotes the firm’s offering, thereby assisting individuals in making brand choices (Kumar et al., 2019). The celebrities’ physical attraction plays a fundamental role in providing an advantage to the markets to promote their brands’ offerings. Customers often buy products based on their likeness. This source of endorsement makes the customers feel that the celebrity’s attractiveness complements the brand’s features. In particular, celebrity attractiveness assists customers’ purchase choices, thus motivating them to buy the product (Onu et al., 2019). In addition, the celebrity attractiveness also makes the advertising appealing and captivating. Given the explanation, the study shows that the celebrity appearance promotes the brand advertisement, thereby influencing the individual’s purchase intention (Lee and Chen, 2021).

Furthermore, building on the source influence, celebrity credibility is a vital component stimulating consumer buying choices. In today’s world, nothing sells like a celebrity’s credibility. The celebrities’ credibility assists the consumer buying intention by esteeming the marketing strategies to support business success (Djafarova and Rushworth, 2017). Given the explanation, the study states that celebrities’ credibility elevates the firms’ marketing activities, thereby influencing impulsive buying behavior (Koay et al., 2021). Besides celebrity credibility, endorsers’ expertise also plays a fundamental role in convincing the customers to buy the product. Customers spend less time planning and rely on the endorser’s expertise to guide their purchase choices. The significant changes brought by the novel marketing strategy have fundamentally made the customers alter their purchasing patterns (Zia et al., 2021). However, in this regard, celebrity expertise plays a fundamental role in fostering the firm’s branding. Expertise refers to an individual’s qualification, skills, knowledge, and competency (Hovland et al., 1953). The leaders’ competence enhances the consumers’ understanding, thus persuading them to buy the product. Based on the statement, the study shows that most of the audience evaluates the advertising message based on the source’s competence (Wang and Scheinbaum, 2018). Therefore, the literature depicts those celebrities with high personal characteristics are perceived to be more persuasive in influencing individual purchases.

Indeed, the above research concludes that celebrity traits profoundly improve consumer purchasing power (Lou and Yuan, 2019). Accordingly, previous literature reveals that in older days, marketers’ used different cues (e.g., store appearance, product packaging, shelf display, and store layout) to influence consumers’ buying behavior (Husnain et al., 2019). However, today, celebrity advertising has considerably grabbed the consumers’ attention, thereby enhancing their buying intention. Indeed, this prior literature makes it essential to understand the role of celebrity personality traits in influencing consumer behavior. Based on this statement, the study suggests that researchers should investigate the role of a celebrity’s characteristics (e.g., trustworthiness, attractiveness, credibility, and expertise) in influencing consumers’ impulse buying and purchase intention (Martensen et al., 2018).

However, against this drop back, this study examines the impact of celebrity characteristics (e.g., trustworthiness, attractiveness, credibility, and expertise) on consumers’ impulsive buying. Significantly, the study objective is to identify the influential factors that predict consumer purchasing intention in the textile fashion industry. Indeed, this study incorporates a suitable conceptual model for evaluating the relationship between the variables. It examines the mediating effect of purchase intention influencing the proposed independence.

Significantly, this study deepens marketers’ knowledge regarding consumers’ impulsive buying and purchase intention. This study model provides valuable information to fashion brands by illustrating the factors that contribute to impulsive buying. As indicated by the prior studies, comprehensive literature had presented on this topic, but this is a novel study that incorporates the mediating role of purchase intention in illustrating consumers’ impulsive behavior in the light of the celebrity endorsement model. Fundamentally, this study opens room for future research in consumer behavior. It is a multidimensional study that provides an in-depth analysis of every variable in a nutshell of consumer buying behavior. Indeed, concerning this persuasive framework, the study findings state that companies should adopt appropriate celebrity endorsement strategies for provoking positive consumer buying behavior.

This study consists of six different sections. Section one introduces the research topic, whereas the subsequent section highlights the study background. The third section presents the research methodology, while the fourth section illustrates the study results. Moreover, the fifth section discusses the research findings in correspondence to the prior literature review, and lastly, the final section concludes the topic by documenting the study results.

## Theoretical Background and Hypothesis Development

### Celebrity Trustworthiness, Impulse Buying Tendency, and Purchase Intention

Notably, the growing importance of celebrity endorsement has gained massive acknowledgment in modern marketing. Accordingly, today, the endorser’s trustworthiness has become a prime component appealing to the global consumer. Celebrities’ trustworthiness plays a significant role in escalating the consumers’ impulsive behavior (Baniya, 2017). The celebrity trustworthiness upgrades the acceptability of the product, thereby evoking the individual’s feeling of conviction. The celebrity trustworthiness enhances the buyers’ believability, thus making the customer involved in the impulse buying behavior. Given the articulation, the study shows that the celebrity attributes (i.e., trustworthiness) influence the individuals’ impulse buying behavior (Munjal, 2020). In the fashion industry, source authenticity (e.g., trustworthiness, honesty) modifies the consumers’ purchase choices. In explaining this notion, the study states that celebrities’ authenticity develops the consumers’ connection with the brand offering, thereby stimulating their impulse buying actions (Chung and Cho, 2017). Moreover, the literature shows that consumers mostly buy the products under the influence of source trustworthiness. Given the articulation, the research reveals that the expert’s truthfulness congruent to the brand offerings significantly escalates impulsive buying behavior (Djafarova and Rushworth, 2017).

Indeed, celebrity endorsement has become a common phenomenon worldwide. Today, with the increasing penetration of modern marketing (i.e., social media), celebrity endorsement has become an engine of business success where celebrity characteristics play a dominant role in enhancing the consumers’ perception regarding the product purchase. The study states that endorsers’ trustworthiness positively affects the individual’s buying intention (Qureshi and Malik, 2017). In particular, celebrities play a profound role in enhancing the consumers’ mindset. An endorser’s trustworthiness is a vital component influencing the consumers’ purchase intention. The increased leaders’ authenticity increases consumers’ understanding regarding the purchase. Based on the statement, the research reveals that celebrity trustworthiness influences consumer perception, thereby elevating optimum purchase intention (Pemberton, 2017). Indeed, the literature concludes that celebrity endorsement has significantly evolved by influencing consumer purchasing behaviors. Therefore, the marketer should adopt innovative marketing strategies to enhance consumer behavior. Given the explanation, the study states that organizations’ investing in endorsers’ qualities (i.e., honesty, trustworthiness) enhances the consumers’ perception, thus increasing the individual willingness to buy the product (Lili et al., 2022). Hence, based on the previous data, we have developed the following hypotheses:

*H1*: Celebrity trustworthiness has a positive and significant impact on impulse buying tendency.

*H2*: Celebrity trustworthiness has a positive and significant impact on purchase intention.

### The Attractiveness of Celebrity Endorser, Impulse Buying Tendency, and Purchase Intention

Undoubtedly, in the era of globalization and competition, numerous industries have advanced their marketing tactics with innovative ideas. The fashion industry is at its boom, which has made the marketers use the celebrities’ attractiveness as a novel idea to grasp the customers’ attention. Individuals are often impressed by the endorsers’ lifestyle and their love for their psychical characteristics. However, today, this fundamental notion has made the brands improve their brand awareness by using celebrity attractiveness as the source of influence. Given the articulation, the study shows that celebrity attractiveness positively stimulates impulse buying intention (Khalid and Ahmed, 2018). Indeed, with the increasing social commerce, celebrity endorsement has become a prime component elevating the impulse buying tendency. The source attractiveness refers to the person’s psychical appearance, lifestyle, and personality. In the advertising world, the celebrity appearance holds a great value in brand promotion. The endorser’s physical attraction appears to promote the brands’ offerings by gaining prestige from the buyers. The study supporting this notion shows that celebrity attractiveness leads to impulse buying intention (Trivedi, 2021). However, it encourages marketers to adopt new trends for promoting the product. The spokesperson’s attractiveness is a unique means to foster the influence of the firm’s advertisement on consumer buying. It persuades the consumers to recognize the brand message, thus recording an increase in buying behavior. In explaining this statement, the study states that celebrity attractiveness alters the consumers’ attention toward the product, ultimately provoking impulsive buying (Lee and Chen, 2021). Altogether, personality attractiveness arouses a sudden buying need in individuals. In this regard, the research states that celebrity attractiveness encourages the individuals” to develop a strong association with the brand, thereby compelling the individual to buy the product (Koay et al., 2021).

In particular, today, the social media spread has widely altered the consumers’ perception under the influence of celebrities’ endorsements. However, this rapid shift has made the companies hire renowned public figures to endorse their products. The celebrity attractiveness increases the individual’s persona, which is essential for enhancing the consumer purchase intention (Kumar et al., 2019; Hang et al., 2022). Celebrity physical attractiveness is an effective marketing strategy that influences customers’ purchasing intention. It boosts the purchasing power of individuals, thus directing their attention toward the product features. Given the articulation, the research states that celebrity attraction yields positive results in customer buying (Arora et al., 2019). Undoubtedly, the celebrity’s attractiveness strengthens the consumers’ purchasing power, behavior, and disposition. Celebrity attractiveness fundamentally appeals to the consumers, thereby increasing consumer involvement. In explaining this notion, the research shows that endorsers’ psychical beauty influences the consumers’ experience and buying intention (Rajasekar, 2018). Undoubtedly, celebrity attractiveness also acts as a vital tool in promoting the brand products. It makes the brand message appealing and captivating. Endorsers play a central role in capturing consumer attention through their personality characteristics. Given the articulation, the study states that the endorsers’ attractiveness maximizes the consumers’ brand preferences, thereby increasing their buying intention (Gauns et al., 2018; Sarfraz et al., 2022a). Hence, with its increased importance, marketers should recognize the role of celebrity attractiveness in enhancing consumers’ purchase intentions. Therefore, based on prior research findings, we have concluded the following hypotheses:

*H3*: Attractiveness of celebrity endorser has a positive and significant impact on impulse buying tendency.

*H4*: Attractiveness of celebrity endorser has a positive and significant impact on purchase intention.

### The Credibility of Celebrity, Impulse Buying Tendency, and Purchase Intention

The use of celebrities in the brand advertisement has made the consumers direct their perception regarding the brand offerings. Accordingly, the literature shows that many firms have hired well-known public figures, influencing consumer behavior. In the advertising world, the endorser’s credibility alters the consumers’ attitude toward the brand’s offering, thus provoking an impulsive buying behavior. In explaining this statement, the study states that celebrity credibility positively affects the consumer mindset, thus influencing impulsive purchase behavior (Chen et al., 2021; Pervez et al., 2022). Undoubtedly, in the fashion industry, the aspects of impulse purchasing have given importance to celebrity credibility. The endorsers’ credibility has profoundly influenced the consumer’s behavior. Accordingly, these days, celebrity credibility has become an important marketing strategy inspiring fashion brands to foster the customers’ impulse buying. The celebrity expertise massively appeals to the buyers. It tends to enhance the customers’ perception of the brand offering. The celebrities’ expertise assists the customer’s cognitive process by making them believe the dynamic qualities of the influencers. This celebrity’s personality inspires the customers to form an emotional attachment with the brand, thus fostering their purchase intention. Given the illustration, the research states that the endorsers’ skills and expertise increase the possibility of customers’ impulsive buying (Hussain, 2020). It is noteworthy that celebrity credibility makes the companies win consumers’ expectations. It is no doubt that celebrity credibility makes the consumer respond positively to the advertisement. Accordingly, the research shows that consumers intend to obey the recommendations of the endorser who owns a high credibility rating and rejects the products that showcase a less credible celebrity (Khanom, 2018). Indeed, the source credibility seems to be a vital component influencing consumer actions.

In particular, the most effective mechanism for driving consumer behavior is the endorsers’ credibility trait. The experts’ credibility provides valuable information to the consumers, thereby boosting the effectiveness of the firms’ marketing. The celebrity with high credibility captures consumers’ attention by enhancing their evaluation process and purchasing intentions. Therefore, drawing on the increasing role of celebrity characteristics, the study states that celebrity credibility fundamentally connects the consumer with the brand, driving their purchase intentions (Burnasheva and Suh, 2022; Sarfraz et al., 2022b). Moreover, the literature indicates that the celebrity in the advertisement strengthens the individual purchasing intention. When the source is credible, the customers alter their attitudes toward the brand, thus ensuring positive purchase intention (Weismueller et al., 2020). This effectiveness of the celebrity personality motivates the customers to buy the product (Singh and Banerjee, 2018). Indeed, the endorsers’ credibility is a powerful stimulus for the consumers’ purchases. Given the illustration, the research shows that it is a mechanism that stimulates the consumers’ cognitive and emotional components, thus assisting the individual in purchase intention (Parmar et al., 2020). Perhaps, the literature concludes that the endorsers’ credibility generates a favorable impact on the consumer impulse buying and purchase intention. Consequently, based on the above literature, we have formulated the following hypotheses:

*H5*: Credibility of celebrity endorser has a positive and significant impact on impulse buying tendency.

*H6*: Credibility of a celebrity endorser has a positive and significant impact on purchase intention.

### Celebrity Expertise, Impulse Buying Tendency, and Purchase Intention

Recently, personality endorsement has been an essential weapon, influencing consumers’ impulse buying tendencies. Previously, consumers used to be inspired by celebrity appearance, attractiveness, and charisma, but in this modern era, consumers have become more inclined toward celebrity expertise, competency, and knowledge. Therefore, today, companies have widely utilized celebrity expertise endorsement to direct consumers’ attention toward impulsive buying. In this regard, the research shows that influencers’ characteristics (e.g., credibility) elevate the consumers’ impulsive buying behavior (Wangshu and Guanhua, 2020). Furthermore, the study states that the celebrities’ competencies inspire the consumers to buy the product recommended by their favorite person (Zafar et al., 2021). In particular, the use of influencers in brand promotion has considerably gained attention over the past decades. Altogether, the celebrities’ skills add value to brand promotion by inevitably persuading the customers to convert their buying thoughts into impulse purchasing behavior.

A celebrity endorsement strategy illustrates that influencers’ expertise plays a significant role in prioritizing the individuals’ purchase choices. The endorsers’ skills, knowledge, and competencies enhance the effectiveness of the individual’s decision-making, thereby promoting positive purchase intention. However, the celebrity endorsement model highlights the significance of the celebrity expertise and states that the perceived expertise of the source inspires the consumers to indulge in rigorous buying behavior. The endorser’s expertise positively influences the customer’s purchase intention (Weismueller et al., 2020). Indeed, the endorser’s knowledge, skill, and experience benefit the consumers by positively influencing their purchase choices. The literature depicts that it is true that celebrities are more convincing when they hold high expertise in persuading the customers’ decision-making. However, in the eyes of the consumers, the celebrity endorser’s expertise is proven to be influential in enhancing consumer buying behavior. In this regard, the study shows that spokespersons’ expertise positively stimulates the individuals’ purchase decisions (Gunawan et al., 2021). Indeed, the celebrity is perceived as influential if he holds the expertise of boosting consumer confidence in the product. In explaining this statement, the researchers state that celebrity expertise has become a significant component in influencing consumers’ purchasing outcomes (Khan, 2020). The influencer’s expertise is an essential aspect in driving customer purchases. Given the explanation, the study shows that celebrity expertise helps individuals make the right buying decision (Anand, 2019). However, it is noteworthy that the celebrity expertise fosters the consumers’ impulse buying and purchase intentions, thereby forming the following relationships:

*H7*: Celebrity expertise has a positive and significant impact on impulse buying tendencies.

*H8*: Celebrity expertise has a positive and significant impact on purchase intention.

### Mediating Role of Purchase Intention

Over the years, various studies have highlighted the factors influencing consumers’ impulsive buying. However, the analysis of these factors shows that consumer purchasing intention has a significant direct effect on consumers’ impulsive behavior. Significantly, advertising has a great capacity to modify consumer behavior. In explaining this notion, the study states that the individual’s purchase intention triggers the individual’s abrupt buying behavior (Ding et al., 2020). In particular, consumers’ impulsive behavior is the outcome of an individual’s psychological desire (Wertenbroch et al., 2020). In this context, the study shows that purchase intention shapes the individuals’ desires (Meena, 2018), substantially leading to impulsive buying.

Indeed, the use of celebrities in promotion campaigns has become the dominant strategy of most brands. The purpose of the organization is to gain consumer attention and acceptability. In this regard, celebrity trustworthiness develops a strong association between consumers and the brand, thereby influencing the consumers’ purchase behavior. Given the articulation, the study shows that celebrity trustworthiness is a significant factor that yields positive purchase intention (Saleem, 2017), thereby encouraging impulsive buying behavior. Similarly, the celebrity attractiveness is also perceived to gain coverage in modern marketing in the context of increased consumer purchase intention. Celebrity likeliness enhances the individuals’ purchase perceptions, thereby accelerating impulsive buying (Gauns et al., 2018). Indeed, the literature indicates that consumers find the celebrity attractiveness to be an effective measure of supporting consumers’ desires and buying behavior (Abbas et al., 2018).

Moreover, the high source credibility also draws consumers’ attention toward impulse buying. In recent years, the companies realizing the importance of source credibility are now hiring celebrities who owns a massive customer base. In explaining this phenomenon, the study shows that celebrity credibility stimulates consumers’ willingness to buy the product (Chin et al., 2020). In the same vein, another research states that celebrity credibility influences the brand image and individuals’ purchase intention (Vidyanata et al., 2018). Additionally, the prior literature also shows that the leaders’ expertise increases the effect of the firms’ message on consumer purchase intention. Celebrity expertise internationalizes consumer purchasing power by investigating its impact on impulsive buying (Khan, 2020).

In particular, consumer purchase intention utilizes celebrity characteristics (e.g., trustworthiness, attractiveness, credibility, expertise) to persuade the customers to involve in impulsive buying behavior. As a result, today, numerous organizations have found using different celebrity personality traits for endorsing the brand. The celerity’s trustworthiness, attraction, credibility, and expertise inspire the customers’ to purchase the product. These notions increase the brand affiliation by forming a deep connection between customers and the brands. Given the articulation, the study states that the influencers’ attractiveness, trustworthiness, expertise, and credibility positively influence the individuals’ impulsive buying intention (Osei-Frimpong et al., 2019). Therefore, based on the prior findings, we propose the following hypotheses:

*H9*: Purchase intention has a positive and significant impact on impulse buying tendency.

*H9(a)*: Purchase intention mediates the relationship between celebrity trustworthiness and impulse buying tendency.

*H9(b)*: Purchase intention mediates the relationship between attractiveness of celebrity endorser and impulse buying tendency.

*H9(c)*: Purchase intention mediates the relationship between the credibility of celebrity endorser and impulse buying tendency.

*H9(d)*: Purchase intention mediates the relationship between celebrity expertise and impulse buying tendency.

**Figure 1:**
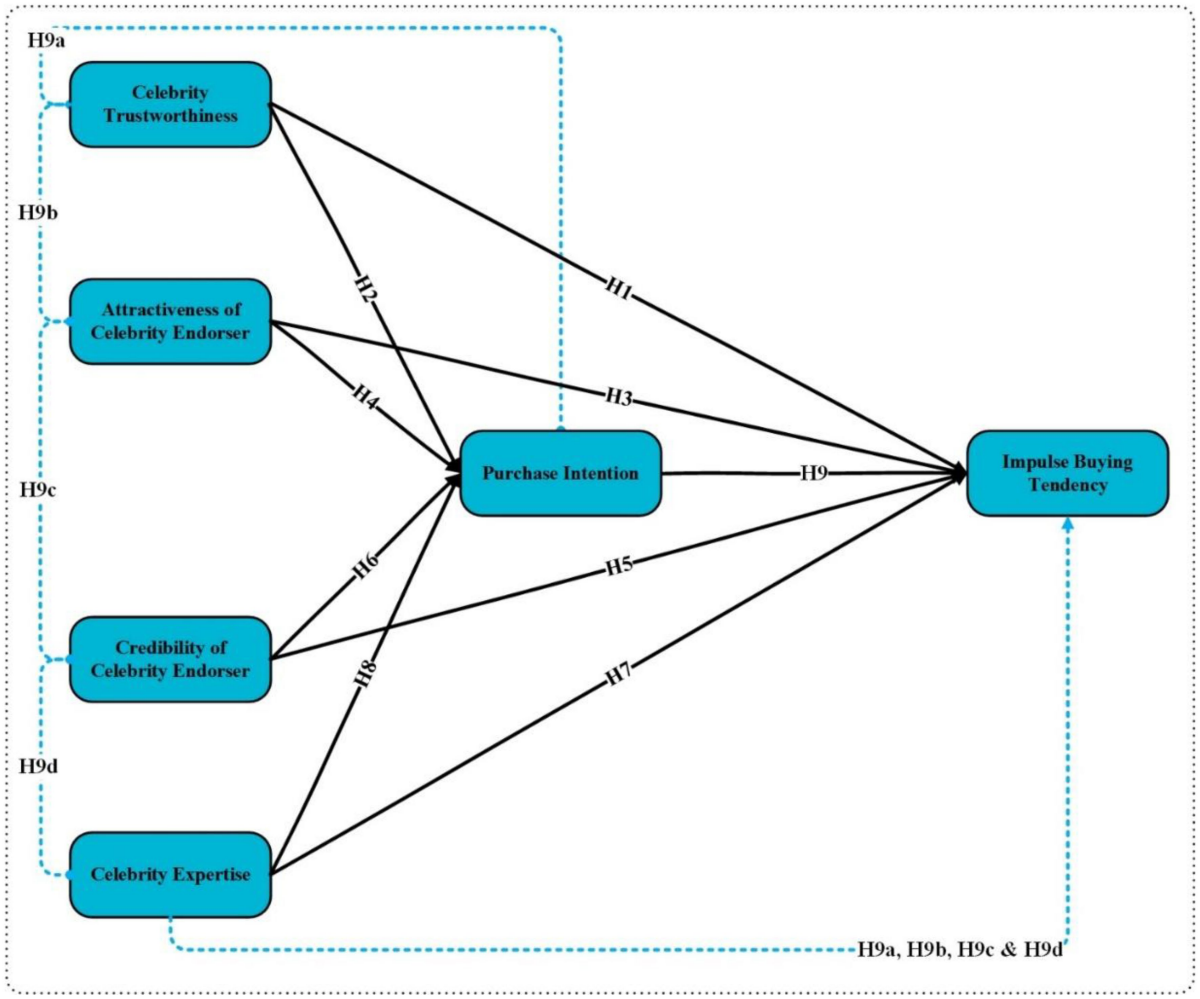
Study framework.

Figure 1 presents the study framework, which includes independent variables (Celebrity Trustworthiness, Attractiveness of Celebrity Endorser, Credibility of Celebrity Endorser, and Celebrity Expertise), mediating variable (Purchase Intention), and dependent variable (Impulse Buying Tendency).

## Methodology

The study’s primary purpose was to measure influencers marketing initiatives on the fast fashion industry by considering the mediating role of purchase intention. The data was collected from the fast fashion industry consumers in China using the convenient-sampling technique. Statistical Package for the Social Sciences (SPSS) software was used for data analysis. Partial least squares structural equation modeling (PLS-SEM) was used to test the study variables’ hypothesis. Four hundred fifty questionnaires were distributed, and 371 valid questionnaires were used for the data analysis. A five-point Likert scale was used for study variables measurement.

### Study Measurement

Celebrity Trustworthiness was measured on the 4-items scale adopted from the study of Wang et al. (2017). The sample items include “I think the advertisements with a trustworthy (dependable, honest, sincere, reliable) endorser receives less negative recall” and “I feel that advertisements with a trustworthy endorser push me to remember that advertisement and the product that is being endorsed.” The attractiveness of celebrity endorsers was measured on the 6-items scale adopted from the studies of Hani et al. (2018). The sample items include “I believe that (Brand Name) endorsed by celebrities are their favorite brands” and “I trust (Brand Name) brands that are endorsed by celebrities.” Celebrity Expertise was measured on the 4-items scale which was adapted from the studies of Wang and Scheinbaum (2018). The Purchase intention was measured on the 5-items scale adopted from the study of Hani et al. (2018), while Impulse buying tendency was measured on the 3-items scale adopted from the study of Bellini et al. (2017). Credibility of Celebrity Endorser was measured on the five items scale adopted from the study of Hani et al. (2018).

## Results and Discussion

Table 1 provides the demographic characteristics of respondents who participate in this study. The survey was conducted under the classifications of Gender, Age, Education, and Marital Status. In the first category of Gender, out of 371 frequency, 187 male and 184 female individuals participated. The participants were further classified age-wise. Forty-eight participants were under the age of 20, 102 falling in the interval of 21–30, 93 in the interval 31–40, 81 in the interval 41–50, and 47 participants were above 50 years of age. Furthermore, 66 participants qualified as intermediate, 138 with a bachelor’s degree, 105 with master’s degree, and 62 participants held M.Phil. or equivalent degrees. The marital status of 184 participants was single, and 187 were married.

**Table 1:** Demographic characteristics.

Items	Frequency (*N* = 371)	(%)
*Gender*		
Male	187	50.4
Female	184	49.6
*Age*		
Under 20	48	12.9
21–30	102	27.5
31–40	93	25.1
41–50	81	21.8
*>50*	47	12.7
*Education*		
Intermediate	66	17.8
Bachelor	138	37.2
Master	105	28.3
MPhil/Others	62	16.7
*Marital Status*		
Single	184	49.6
Married	187	50.4

### Common Method Bias

This research applied the common method bias using Harman’s single-factor approach. The variance extracted by one single factor is 13.275% which is less than 50%, indicating no common method bias in this study (Podsakoff et al., 2003).

Table 2 shows the reliability and validity analysis values of celebrity trustworthiness (α-0.894, AVE-0.697), attractiveness of celebrity endorser (α-0.924, AVE-0.670), credibility of celebrity endorser (α-0.917, AVE-0.688), celebrity expertise (α-0.866, AVE-0.661), purchase Intention (α-0.915, AVE-0.682) and impulse buying tendency (α-0.915, AVE-0.682).

**Table 2:** Reliability and validity analysis.

Construct	Items	Loading	*α*	CR	AVE
Attractiveness of Celebrity Endorser	ACE_1	0.867	0.924	0.924	0.670
	ACE_2	0.825			
	ACE_3	0.794			
	ACE_4	0.752			
	ACE_5	0.812			
	ACE_6	0.856			
Celebrity Trustworthiness	CTW_1	0.833	0.894	0.894	0.679
	CTW_2	0.801			
	CTW_3	0.825			
	CTW_4	0.836			
Credibility of Celebrity Endorser	CCE_1	0.782	0.917	0.917	0.688
	CCE_2	0.860			
	CCE_3	0.842			
	CCE_4	0.877			
	CCE_5	0.783			
Celebrity Expertise	CE_1	0.788	0.886	0.886	0.661
	CE_2	0.810			
	CE_3	0.756			
	CE_4	0.892			
Purchase Intention	PI_1	0.789	0.915	0.915	0.682
	PI_2	0.853			
	PI_3	0.834			
	PI_4	0.831			
	PI_5	0.821			
Impulse Buying Tendency	IBT_1	0.832	0.860	0.860	0.672
	IBT_2	0.802			
	IBT_3	0.824			

**Figure 2:**
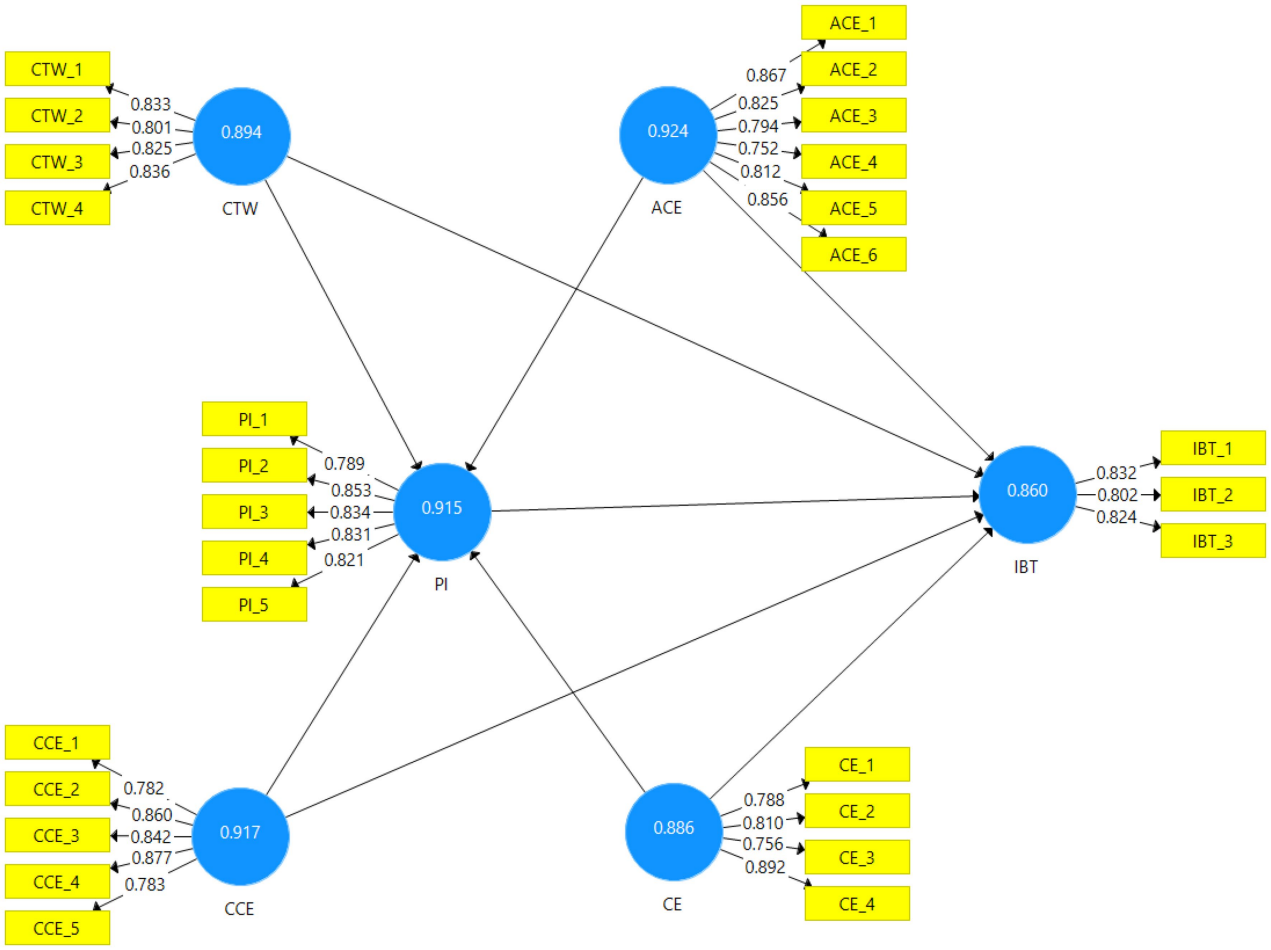
Graphical representation of assessment of measurement model.

Table 3 presents discriminant validity results by using the Fornel Larcker and HTMT method. The discriminant validity value of attractiveness of celebrity endorser value is 0.819, the credibility of celebrity endorser (0.830), celebrity expertise (0.813), celebrity trustworthiness (0.824), impulse buying tendency (0.820), and purchase intention (0.826). Figure 2 shows the graphical representation of the assessment of the measurement model.

**Table 3:** Discriminant validity analysis (Fornel Larcker and HTMT).

S. No.	Constructs	1	2	3	4	5	6
1.	Attractiveness of Celebrity Endorser	**0.819**	0.647	0.615	0.648	0.675	0.653
2.	Credibility of Celebrity Endorser	0.646	**0.830**	0.628	0.648	0.669	0.646
3.	Celebrity Expertise	0.613	0.627	**0.813**	0.629	0.666	0.631
4.	Celebrity Trustworthiness	0.647	0.649	0.628	**0.824**	0.662	0.643
5.	Impulse Buying Tendency	0.676	0.670	0.666	0.662	**0.820**	0.685
6.	Purchase Intention	0.654	0.647	0.633	0.643	0.685	**0.826**

**Figure 3:**
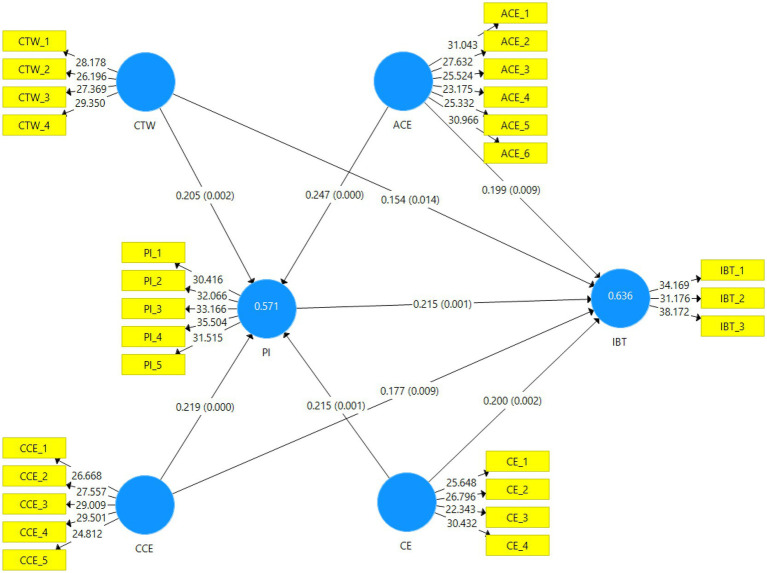
Graphical representation of the structural model.

Table 4 shows the direct relation between celebrity trustworthiness, attractiveness of celebrity endorser, the credibility of a celebrity endorser, celebrity expertise, purchase intention, and impulse buying tendency. Hypothesis H1 (Celebrity trustworthiness has a positive and significant impact on impulse buying tendency) was accepted at the beta value of 0.154. Hypothesis H2 (Celebrity trustworthiness has a positive and significant impact on purchase intention) was accepted at the beta value of 0.205. Hypothesis H3 (Attractiveness of celebrity endorser has a positive and significant impact on impulse buying tendency) was accepted at the beta value of 0.199. Hypothesis H4 (Attractiveness of celebrity endorser has a positive and significant impact on purchase intention) was accepted at the beta value of 0.247. Hypothesis H5 (Credibility of celebrity endorser has a positive and significant impact on impulse buying tendency) was accepted at the beta value of 0.177. Hypothesis H6 (Credibility of celebrity endorser has a positive and significant impact on purchase intention) was accepted at the beta value of 0.219. Hypothesis H7 (Celebrity expertise has a positive and significant impact on impulse buying tendency) was accepted at the beta value of 0.200. Hypothesis H8 (Celebrity Expertise Has Positive and Significant Impact on Purchase Intention) was accepted at the beta value of 0.215. Hypothesis H9 (Purchase Intention has a positive and significant impact on purchase intention) was accepted at the beta value of 0.215. Hypothesis H9(a) states that purchase intention mediates the relationship between celebrity trustworthiness and impulse buying tendency. The hypothesis H9(a) was accepted at the beta value of 0.044. Hypothesis H9(b) states that purchase intention mediates the relationship between the attractiveness of celebrity endorsers and impulse buying tendencies. The hypothesis H9(b) was accepted at the beta value of 0.053. Hypothesis H9(c) states that purchase intention mediates the relationship between the credibility of celebrity endorsers and impulse buying tendencies. The hypothesis H9(c) was accepted at the beta value of 0.047. According to Hypothesis H9(d), purchase intention mediates the relationship between celebrity expertise and impulse buying tendency. The hypothesis H9(d) was accepted at the beta value of 0.046 (see Table 5). Figure 3 is a graphical representation of the structural model.

**Table 4:** Hypotheses testing direct effect.

Hypothesis	Direct relationships	Std. *beta*	Std. error	*T*	*P*
H1	CTW → IBT	0.154	0.063	2.461	^*^
H2	CTW → PI	0.205	0.066	3.099	^**^
H3	ACE → IBT	0.199	0.076	2.629	^**^
H4	ACE → PI	0.247	0.057	4.304	^***^
H5	CCE → IBT	0.177	0.067	2.629	^**^
H6	CCE → PI	0.219	0.063	3.502	^***^
H7	CE → IBT	0.200	0.064	3.106	^**^
H8	CE → PI	0.215	0.063	3.385	^**^
H9	PI → IBT	0.215	0.067	3.224	^**^

* Indicates significant path: *p* < 0.05.

** Indicates significant path: *p* < 0.01.

*** Indicates significant path: *p* < 0.001.

**Table 5:** Hypotheses testing mediation effect.

Hypothesis	Indirect relationships	Std. *beta*	Std. error	*T*	*P*
H9a	CTW → PI → IBT	0.044	0.021	2.089	^*^
H9b	ACE → PI → IBT	0.053	0.021	2.525	^*^
H9c	CCE → PI → IBT	0.047	0.021	2.276	^*^
H9d	CE → PI → IBT	0.046	0.022	2.106	^*^

NS, not significant.

* Indicates significant path: *p* < 0.05.

In this modern advertising period, the international market flooded with different brands has elevated the need for innovative marketing strategies. However, in this regard, the celebrity endorsement strategy has profoundly been realized as the most influential icon accelerating individuals’ purchasing behavior. The celebrity harmonizes the firms’ marketing activities through their effective personality traits. Their profound qualities (e.g., trustworthiness, attractiveness, credibility, and expertise) inevitably influence the individual’s impulsive buying. Over the years, these personality factors have grabbed the consumers’ attention, substantially accelerating the individuals’ impulsive buying tendency. In particular, the celebrity characteristics lead the marketers to fulfill the needs of the consumers by potentially realizing an urgent urge for the product. As a result, today, companies are massively investing in spokesperson characteristics for elevating positive consumer behavior.

## Conclusion

Consumer purchasing intentions have intensified due to the increasing concept of celebrity endorsement. Celebrity characteristics endorsement has become a global trend, thus accelerating the consumers’ impulsive buying behavior. The study chooses the textile fashion industry for investigation. The literature shows that fashion purchases have become the norm, with most items brought impulsively. Accordingly, the study concludes that the celebrity characteristics (i.e., trustworthiness, attractiveness, credibility, and expertise) positively influence the impulsive buying tendency and purchase intention. It also confirms a significant mediating role of purchase intention in fostering consumers’ impulsive buying. Undoubtedly, this study holds great significance for the market experts, thus recommending them to improve marketing activities.

The results reveal that the study outcomes are beneficial for future research in the area of consumer buying behavior. In particular, the study presents a significant opportunity for advertisers to understand the novel marketing strategies for capturing consumer attention. However, it provides a new perspective to the researchers, marketers, and policymakers regarding the impulsive buying of fashion brands in the light of the celebrity endorsement model.

## Data Availability Statement

The raw data supporting the conclusions of this article will be made available by the authors, without undue reservation.

## Ethics Statement

Ethical review and approval was not required for the study on human participants in accordance with the local legislation and institutional requirements. The patients/participants provided their written informed consent to participate in this study.

## Author Contributions

The author confirms being the sole contributor of this work and has approved it for publication.

## Conflict of Interest

The author declares that the research was conducted in the absence of any commercial or financial relationships that could be construed as a potential conflict of interest.

## Publisher’s Note

All claims expressed in this article are solely those of the authors and do not necessarily represent those of their affiliated organizations, or those of the publisher, the editors and the reviewers. Any product that may be evaluated in this article, or claim that may be made by its manufacturer, is not guaranteed or endorsed by the publisher.
